# Vascular *microRNA-204* is remotely governed by the microbiome and impairs endothelium-dependent vasorelaxation by downregulating Sirtuin1

**DOI:** 10.1038/ncomms12565

**Published:** 2016-09-02

**Authors:** Ajit Vikram, Young-Rae Kim, Santosh Kumar, Qiuxia Li, Modar Kassan, Julia S. Jacobs, Kaikobad Irani

**Affiliations:** 1 Cardiovascular Division, Department of Medicine, University of Iowa Carver College of Medicine, Iowa City, Iowa 52242, USA; 2 Abboud Cardiovascular Research Center, University of Iowa Carver College of Medicine, Iowa City, Iowa 52242, USA; 3 Fraternal Order of Eagles Diabetes Research Center, University of Iowa, Iowa City, Iowa 52242, USA; 4 Pappajohn Biomedical Institute, University of Iowa, Iowa City, Iowa 52242, USA; 5 Heart and Vascular Center, University of Iowa Hospitals and Clinics, Iowa City, Iowa 52242, USA

## Abstract

Gut microbiota promotes atherosclerosis, and vascular endothelial dysfunction, signalled by impaired endothelium-dependent vasorelaxation, is an early marker of atherosclerosis. Here we show that vascular *microRNA-204* (*miR-204*) expression is remotely regulated by the microbiome, and impairs endothelial function by targeting the Sirtuin1 lysine deacetylase (*Sirt1*). *MiR-204* is downregulated, while Sirt1 is upregulated, in aortas of germ-free mice. Suppression of gut microbiome with broad-spectrum antibiotics decreases *miR-204*, increases Sirt1 and bioavailable vascular nitric oxide, and improves endothelium-dependent vasorelaxation in mouse aortas. Antibiotics curtail aortic *miR-204* upregulation, and rescue decline of aortic Sirt1 and endothelium-dependent vasorelaxation, triggered by high-fat diet feeding. Improvement of endothelium-dependent vasorelaxation by antibiotics is lost in mice lacking endothelial Sirt1. Systemic antagonism of *miR-204* rescues impaired endothelium-dependent vasorelaxation and vascular Sirt1, and decreases vascular inflammation induced by high-fat diet. These findings reveal a gut microbe-vascular microRNA–Sirtuin1 nexus that leads to endothelial dysfunction.

## Abstract (web summary)

Commensal bacteria, the vast majority of which reside in the gut, are involved in development of many diseases, including atherosclerotic vascular disease. Here the authors show that these bacteria remotely increase expression of vascular *microRNA-204*, which targets Sirt1 in the endothelium to impair endothelial function.

## Graphical abstract

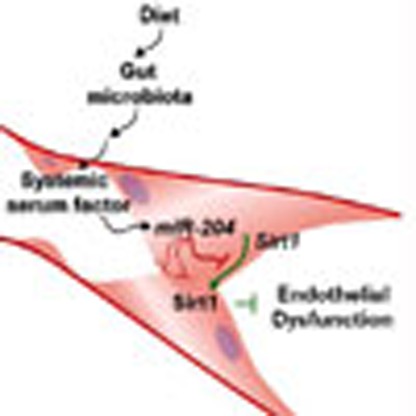

The gut flora, consisting of trillions of microbes, plays an essential role in dietary energy harvest. The gut microbiome has been implicated in the pathogenesis of metabolic disorders such as diabetes^1^ and obesity^2^. The microbiome can also contribute to the development of atherosclerotic vascular disease^3^,^4^,^5^,^6^. Metabolites of dietary phospholipids and nutrients produced by gut flora and found in the systemic circulation contribute to vascular inflammation and atherosclerosis^3^,^5^. On the other hand, the microbiome also participates in retarding atherosclerosis. Gut bacteria generate metabolites of a class of dietary flavonoids that decrease atherosclerotic burden by stimulating reverse cholesterol transport in macrophages^7^.

Endothelial dysfunction, manifested as impaired endothelium-dependent vasorelaxation and vascular inflammation, is a precursor and strong predictor of atherosclerosis^8^. Sirtuin1 (Sirt1) is a class III histone deacetylase expressed in cell types comprising the vascular wall that stimulates endothelial nitric oxide (NO) by deacetylating endothelial nitric oxide synthase (eNOS), promotes endothelium-dependent vasorelaxation, and protects against endothelial dysfunction and atherosclerosis^9^,^10^. Sirt1 expression is governed by microRNAs^11^. For example, *miR-217* and *miR-212*, both of which directly target Sirt1, promote endothelial cell senescence and suppress *in vitro* angiogenesis^12^,^13^. We hypothesized and prove that the expression of vascular Sirt1 is an important determinant of gut microbiome-dependent endothelial function, and vascular *miR-204*, one of the microRNAs whose expression is remotely controlled by the microbiome, targets endothelial Sirt1 leading to endothelial dysfunction.

## Results

### Microbiome stimulates the expression of vascular *miR-204*

**Figure 1:**
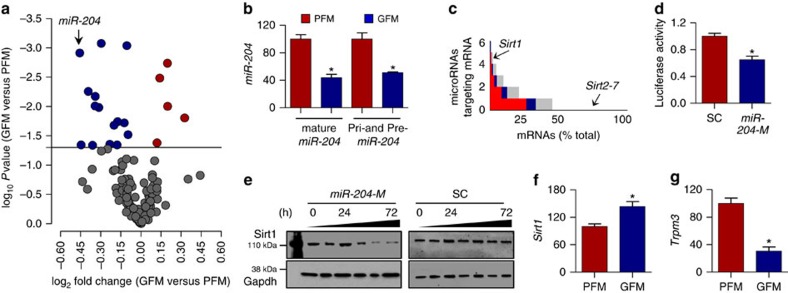
*MiR-204* is downregulated and *Sirt1* is upregulated in aortas of GFM. (**a**) Volcano plot of differentially expressed microRNAs in aortas of germ-free mice (GFM). Difference in the expression of aortic microRNAs between GFM and pathogen-free mice (PFM) is plotted on the *x* axis (log_2_ scale), and significance is plotted on the *y* axis (log_10_ scale). Upregulated or downregulated microRNAs are indicated in red and blue, respectively. *n*=3 mice for each microRNA. (**b**) Decreased mature and precursor *miR-204* expression in aortas of GFM by qPCR (shown as % of PFM). *n*=4 for each group, *P<0.05 versus PFM. (**c**) *In silico* analysis of mRNAs targeted by microRNAs that were downregulated by nCounter assay in aortas of GFM. Number of downregulated microRNAs targeting a specific mRNA is shown on the *y* axis and % of total mRNA is shown on the *x* axis. MicroRNA-SVR score is colour-coded with threshold<−0.50 in grey, <−0.75 in blue and<−0.90 in red. Lower SVR score indicates stronger predicted microRNA–mRNA interaction. *Sirt1* is targeted by microRNAs downregulated in GFM aortas, and based on the number of microRNAs targeting a specific gene it was predicted to be in top 0.5% of putatively regulated genes. (**d**) *MiR-204* mimic (*miR-204-M*) decreases *Sirt1* 3′-UTR luciferase reporter activity. SC, scrambled control microRNA. *P<0.05 versus SC, *n*=5 independent experiments. (**e**) *MiR-204-M* (20 nM) decreases Sirt1 expression in human umbilical vein endothelial cells (HUVECs) in a time-dependent manner. Representative of three independent experiments is shown. (**f**,**g**) Increased *Sirt1* (**f**) and decreased *Trpm3* (**g**) mRNA expression in aortas of GFM (shown as % of PFM). *n*=4 for each group, *P<0.05 versus PFM. Independent sample *t*-test was used. Data shown as mean and error bar represents s.e.m.

Initially, we sought to identify the effect of the microbiota on vascular microRNA expression profiles. Unbiased microRNA nCounter expression arrays were performed and compared in aortas of microbiota-free germ-free mice (GFM) and control pathogen-free mice (PFM). Of the 578 microRNAs assayed, 15 were found to be significantly downregulated, while 5 were upregulated in aortas of GFM compared with PFM (Fig. 1a; Supplementary Fig. 1a). Global microRNA expression, and expression of the most abundant vascular microRNAs, did not differ between GFM and PFM (Supplementary Fig. 1b,c). Similarly, aortic expression of microRNA processing enzymes Dicer and Drosha were not altered in germ-free conditions (Supplementary Fig. 1d). Among the microRNAs downregulated by nCounter array in GFM aortas (Supplementary Fig. 2d), *miR-204* and *miR-148a* were confirmed by quantitative PCR (qPCR), with a strong trend for *miR-9* (Supplementary Fig. 2e). Since *miR-204* was the most robustly downregulated microRNA, we henceforth focused our attention on it. Decrease of *miR-204* in GFM aortas (Fig. 1b) was validated using different internal controls (Supplementary Fig. 2c) and expression of *miR-204* precursors in the aorta followed a trend similar to that of the mature microRNA (Fig. 1b). *In situ* hybridization showed that *miR-204* is expressed in both the endothelium and tunica media of mouse aortas (Supplementary Fig. 3).

### Sirt1 is targeted by *miR-204* and is upregulated in GFM

Computational microRNA–mRNA algorithms predicted *Sirt1* to be in the top 100 genes (0.5 percentile) targeted by the conserved microRNAs downregulated by nCounter array in aortas of GFM (Fig. 1c). Among these microRNAs, *miR-204*, *miR-9*, *miR-142-3p* and *miR-199a-5p* have been experimentally shown to downregulate Sirt1 (refs 14, 15, 16). Focusing on *miR-204*, we confirmed that it targets Sirt1 in vascular endothelial cells using *miR-204* modulators (mimic, inhibitor) and Sirt1 3′-untranslated region (UTR) reporter assay (Fig. 1d,e; Supplementary Fig. 4). Furthermore, *Sirt1* was upregulated in several tissues, including the aortas (Fig. 1f), skeletal muscle and lungs, but not in the heart and liver of GFM (Supplementary Fig. 2f). Because *miR-204* is in intron 6 of the *Trpm3* gene located on chromosome 9q21.12, and is co-expressed with *Trpm3* (ref. 17), we also measured *Trpm3* expression. Paralleling *miR-204* expression, *Trpm3* was downregulated in GFM aortas (Fig. 1g).

### Antibiotics decrease *miR-204* and increase vascular Sirt1

**Figure 2:**
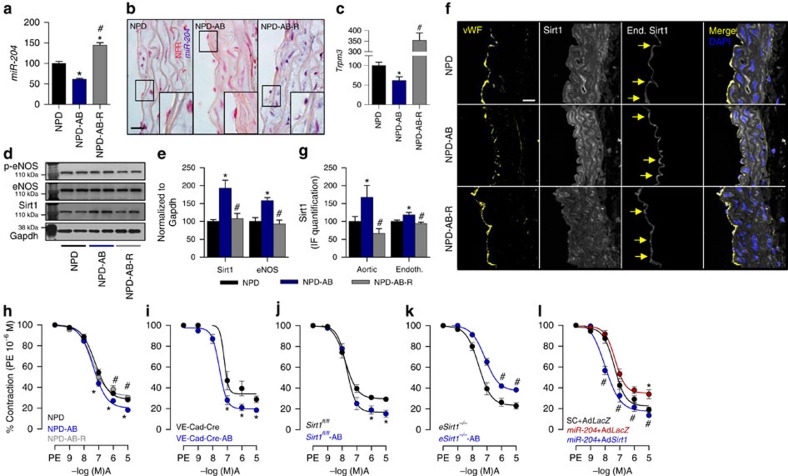
Antibiotics improve vasorelaxation via endothelial Sirt1. (**a**–**c**) Gut microbiota suppression in mice with broad-spectrum antibiotics downregulates aortic *miR-204* (**a**: qPCR; **b**: *in situ* hybridization at × 100 magnification with *miR-204* shown in purple with red nuclear counterstain, Scale bar; 20 μm) and *Trpm3* (**c**). All data are shown as % of NPD. *n*=6 for NPD and NPD-AB, *n*=4 for NPD-AB-R. *P<0.05 versus NPD, ^#^*P*<0.05 versus NPD-AB. (**d**,**e**) Gut microbiome suppression in mice with broad-spectrum antibiotics upregulates aortic Sirt1 and eNOS expression. All data are shown as % of NPD. *n*=3 for NPD and NPD-AB, *n*=4 for NPD-AB-R. *P<0.05 versus NPD, ^#^*P*<0.05 versus NPD-AB. (**f**) Immunofluorescence of mouse aortic sections showing upregulation of total aortic and endothelial Sirt1 expression with antibiotics (magnification × 63, Scale bar; 20 μm). (**g**) Quantification of total aortic (*n*=4 sections) and endothelial (*n*=15 endothelial cells) Sirt1 immunostaining in **f**. (**h**,**i**) Gut microbiota suppression in mice with antibiotics improves endothelium-dependent vasorelaxation in wild-type C57Bl/6 (**h**), *VE-Cad-Cre* (**i**) and *Sirt1*^*fl/fl*^ (**j**) mice. NPD: *n*=10, NPD-AB: *n*=10, NPD-AB-R: *n*=8. *P<0.05 versus NPD, ^#^*P*<0.05 versus NPD-AB. *Sirt1*^*fl/fl*^: *n*=10, *Sirt1*^*fl/fl*^-AB: *n*=20; *P<0.05 versus *Sirt1*^*fl/fl*^. *VE-Cad-Cre*: *n*=5, *VE-Cad-Cre*-AB: *n*=9, *P<0.05 versus *VE-Cad-Cre*. (**k**) Gut microbiome suppression with antibiotics does not improve aortic endothelium-dependent vasorelaxation in *eSirt*^*−/−*^ mice. *eSirt1*^*−*/*−*^: *n*=14, *eSirt1*^*−*/*−*^AB: *n*=15. ^#^*P*<0.05 versus *eSirt1*^*−/−*^. (**l**) Adenovirus-mediated overexpression of *Sirt1* lacking 3′-UTR rescues *miR-204* mimic-induced impairment of endothelium-dependent vasorelaxation *ex vivo.* SC-Ad*LacZ*: *n*=5; *miR-204*-Ad*LacZ*: *n*=6; *miR-204*-Ad*Sirt1*: *n*=6. Ad*LacZ*, control adenovirus-expressing *E. coli LacZ*; Ad*Sirt1*, adenovirus-expressing *Sirt1*; SC, scrambled control microRNA. *P<0.05 versus SC-AdLacZ, ^#^*P*<0.05 versus *miR-204*-Ad*LacZ*. A, acetylcholine; AB, antibiotics; *eSirt1*^*−/−*^, mice conditionally lacking endothelial Sirt1; IF, immunofluorescence; NFR, nuclear fast red; NPD, normal pellet diet; PE, phenylephrine; R, stoppage of antibiotics and recolonization; *Sirt1*^*fl/fl*^, control Sirt1 floxed mice. *n* represents the number of aortic rings in **h**–**l**. Independent sample *t*-test was used. Data shown as mean and error bar represents s.e.m.

We next asked whether suppressing gut flora biomass with broad-spectrum oral antibiotics results in changes in vascular *miR-204* and Sirt1 expression similar to that observed in GFM. Wild-type C57Bl/6 mice isolated from the colony, housed in separate cages and treated with antibiotics in drinking water for 6 weeks were slightly leaner than their untreated counterparts but had similar plasma lipids (Supplementary Fig. 5a,b). Mice on antibiotics had markedly lower faecal microbial load (Supplementary Fig. 6). Administration of antibiotics decreased aortic *miR-204* and *Trpm3* (Fig. 2a–c), while increasing aortic Sirt1 (Fig. 2d–g). Discontinuation of antibiotics and co-housing with mice not on antibiotics reversed changes in aortic expression of *miR-204*, *Trpm3* and Sirt1 (Fig. 2a–g). Immunohistochemistry showed that total aortic Sirt1 as well as endothelial Sirt1 was upregulated in mice receiving antibiotics (Fig. 2d–g).

### Antibiotics promote endothelial function via Sirt1

Because of the important role of Sirt1 in eNOS signalling, we questioned whether the gut microbiome stimulates vascular NO and endothelium-dependent vasorelaxation. Antibiotic administration to mice upregulated eNOS (Fig. 2d,e), and improved endothelium-dependent vasorelaxation (Fig. 2h), in mouse aortas. Repopulation of the microbiome by discontinuation of antibiotics and co-habitation with mice not on antibiotics reversed changes in aortic expression of eNOS and decreased endothelium-dependent vasorelaxation (Fig. 2d,e,h). To determine the role of endothelial Sirt1 in the microbiota-vascular axis, we examined the effect of antibiotics on endothelium-dependent vasorelaxation in mice with conditional deletion of endothelial Sirt1 (*eSirt1*^*−/−*^). In contrast to control wild-type, *Sirt1*^*fl/fl*^ and *VE-Cad-Cre* mice, antibiotics paradoxically impaired endothelium-dependent vasorelaxation in *eSirt1*^*−/−*^ mice (Fig. 2h–k), showing an obligatory role of endothelial Sirt1 in improved vasorelaxation achieved by suppressing the microbiome.

### Sirt1 rescues *miR-204*-induced endothelial dysfunction

To establish a causal role for *miR-204*-induced downregulation of Sirt1 in endothelial dysfunction, *miR-204* mimic was transfected into mouse aortas *ex vivo* resulting in robust upregulation of aortic *miR-204* (Supplementary Fig. 7a). *MiR-204* mimic suppressed endothelial Sirt1 (Supplementary Fig. 7b) and decreased endothelium-dependent vasorelaxation (Fig. 2l). This decrease in endothelium-dependent vasorelaxation was rescued by adenoviral-mediated reconstitution of endothelial Sirt1 (lacking the 3′-UTR) in mouse aortas (Fig. 2l; Supplementary Fig. 7b), indicating that *miR-204* mimic impairs endothelial function, at least in part, by suppressing Sirt1 expression.

### Diet-microbiome interaction governs *miR-204* and Sirt1

**Figure 3:**
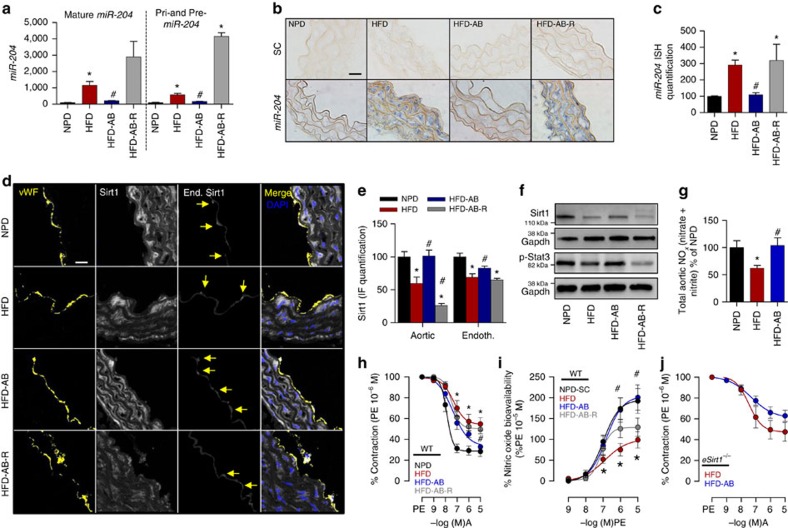
Antibiotics rescue endothelial Stat3 and Sirt1 in high-fat diet-induced vascular dysfunction. (**a**–**c**) Suppression of gut microbiota with broad-spectrum antibiotics inhibits HFD-induced aortic *miR-204*. (**a**) qPCR for mature and precursor *miR-204* in mouse aortas. *n*=4 for NPD and HFD-AB-R, *n*=6 for HFD and HFD-AB. (**b**) *In situ* hybridization for *miR-204* in mouse aortas (blue, magnification × 100, Scale bar; 20 μm). (**c**) Quantification of miR-204 in **b**. *n*=5 images from different regions of aortic sections. (**d**–**f**) Suppression of gut microbiome with antibiotics prevents HFD-induced downregulation of aortic Sirt1 and Stat3 signalling. (**d**) Immunofluorescence for Sirt1 in mouse aortic sections (magnification × 63, Scale bar; 20 μm). (**e**) Quantification of total aortic (*n*=4 sections) and endothelial (*n*=15 endothelial cells) Sirt1 immunostaining in **d**. (**f**) Western blot for Sirt1 and phospho-Stat3 in mouse aortas. Immunoblots were done with pooled aortic samples; *n*=4 for NPD and HFD-AB-R and *n*=6 for HFD and HFD-AB. (**g**) Suppression of gut microbiome rescues HFD-induced decrease in total aortic NO_*x*_ [nitrate+nitrite]. NPD: *n*=9, HFD: *n*=6, HFD-AB: *n*=6. (**h**,**i**) Suppression of gut microbiota rescues HFD-induced reduction of endothelium-dependent vasorelaxation and nitric oxide bioavailability. NPD: *n*=10, HFD: *n*=12, HFD-AB: *n*=10, HFD-AB-R: *n*=10. (**j**) Suppression of gut microbiota does not rescue HFD-induced impairment of endothelium-dependent vasorelaxation in *eSirt1*^*−/−*^ mice. *eSirt1*^*−/−*^ HFD: *n*=6, *eSirt1*^*−/−*^HFD-AB: *n*=12. *P<0.05 versus NPD. ^#^*P*<0.05 versus HFD. A, acetylcholine; AB, antibiotics; *eSirt1*^*−/−*^, mice conditionally lacking endothelial Sirt1; HFD, high-fat diet; NPD, normal pellet diet; PE, phenylephrine; R, stoppage of antibiotics and recolonization. *n* represents the number of aortic rings in **h**–**j**. IF, immunofluorescence. Independent sample *t*-test was used. Data shown as mean and error bar represents s.e.m.

We then questioned whether diet-induced endothelial dysfunction is also dependent on the microbiome and triggered by *miR-204*-mediated downregulation of vascular Sirt1. To address this, we first measured aortic expression of Sirt1 and *miR-204* in a high-fat diet (HFD) feeding model of endothelial dysfunction. HFD feeding of C57Bl/6 mice for 8 weeks upregulated aortic *miR-204* and its precursors (Fig. 3a–c), and downregulated total aortic and endothelial Sirt1 (Fig. 3d–f). Antibiotics administered during the last 6 weeks of the HFD feeding period decreased aortic *miR-204* to basal levels (Fig. 3a–c), and restored aortic and endothelial Sirt1 (Fig. 3d–f). Discontinuation of antibiotics and co-habitation of the mice with littermates not on antibiotics for 2 weeks reversed changes in aortic *miR-204* (Fig. 3a–c) and Sirt1 (Fig. 3d–f).

### Antibiotics reverse HFD-induced endothelial dysfunction via Sirt1

HFD feeding impaired endothelium-dependent vasorelaxation (Fig. 3h), and reduced bioavailable vascular NO, in mouse aortas (Fig. 3i). Broad-spectrum antibiotics rescued this decline in endothelium-dependent vasorelaxation (Fig. 3h) and bioavailable NO (Fig. 3i). Cessation of antibiotics and re-housing the mice with those not on antibiotics negated improvement in endothelium-dependent vasorelaxation and bioavailable NO (Fig. 3h,i). Importantly, rescue of endothelium-dependent vasorelaxation by antibiotics was lost in *eSirt1*^*−/−*^ mice (Fig. 3j), underscoring the role of endothelial Sirt1 in mediating the beneficial effect of gut microbiota suppression on vascular endothelial function.

### Antibiotics promote endothelial Stat3 signalling

We next sought to understand the molecular mechanism responsible for the regulation of vascular *miR-204* expression by the gut microbiome. The transcription factor Stat3 represses *miR-204* expression in vascular smooth muscle cells and pancreatic beta cells^18^,^19^. We therefore explored the role of Stat3 signalling in regulation of vascular *miR-204* by the gut microbiome. Inhibition of Stat3 signalling in endothelial cells with a dominant-negative Stat3 construct stimulated *miR-204* expression (Supplementary Fig. 8a). Moreover, activated (phosphorylated) Stat3 was diminished in aortas of mice on HFD (Fig. 3f; Supplementary Fig. 8b,c). Suppression of the microbiome with antibiotics rescued aortic Stat3 signalling, while reconstitution of the gut flora diminished phospho-Stat3 (Fig. 3f; Supplementary Fig. 8b,c). Importantly, phosphorylation of Stat3, and changes in its phosphorylation with HFD and antibiotics, was seen principally in the endothelium (Supplementary Fig. 8b,c), indicating that endothelial Stat3 signalling is an important target of the gut microbiome.

### *MiR-204* mediates HFD-induced endothelial dysfunction

**Figure 4:**
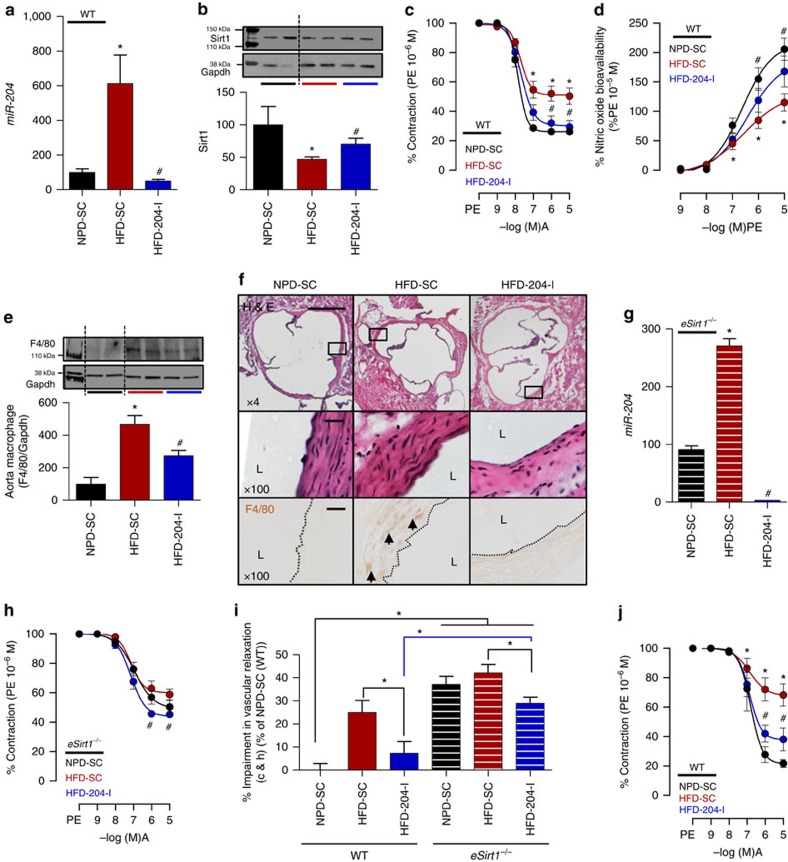
*MiR-204* mediates high-fat diet-induced vascular dysfunction and inflammation. (**a**) Systemic delivery of *miR-204* inhibitor in HFD-fed mice (**a**) prevents upregulation of aortic *miR-204*, (**b**) downregulation of aortic Sirt1 and (**c**,**d**) rescues endothelium-dependent vasorelaxation and bioavailable vascular NO. *n*=4 for NPD-SC, *n*=6 for HFD-SC and HFD-204-I in **a**; *n*=4 for NPD-SC, *n*=6 for HFD-SC and HFD-204-I in **b**; NPD-SC: *n*=15, HFD-SC: *n*=16, HFD-204-I: *n*=18 in **c**,**d**. *n* represents number of aortic rings. (**e**,**f**) Systemic *in vivo* delivery of *miR-204* inhibitor suppresses HFD-induced vascular inflammation. Expression of macrophage marker f4/80 was assessed by immunoblot in aorta (**e**) and by immunohistochemistry (**f**) in aortic roots of mice (magnification × 4 or × 100; scale bars, 500 μm (× 4), 20 μm (× 100)). Adjacent sections of aortic root sections were stained with haematoxylin and eosin (magnification × 4 and 100). *n*=4 for NPD-SC, *n*=6 for HFD-SC and HFD-204-I, *P<0.05 versus NPD-SC. ^#^*P*<0.05 versus HFD-SC. HFD, high-fat diet; NPD, normal pellet diet; SC, scrambled control microRNA; 204-I, miR-204 inhibitor. (**g**–**i**) Inhibition of *miR-204* with HFD feeding improves endothelium-dependent vasorelaxation to a lesser degree in *eSirt1*^*−/−*^ mice compared with wild-type mice. (**g**) Aortic expression of *miR-204* in *eSirt1*^*−/−*^ mice with HFD and with *miR-204* inhibitor infusion. RT–PCR was done with pooled aortic samples. (**h**) Systemic delivery of *miR-204* inhibitor partially improves endothelium-dependent vasorelaxation induced in *eSirt1*^*−/−*^ mice on HFD. *n* represents the number of aortic rings. NPD-SC: *n*=13, HFD-SC: *n*=12, HFD-204-I: *n*=9. (**i**) Per cent impairment in endothelium-dependent vascular relaxation in **c**,**h**. (**j**) *Ex vivo* inhibition of *miR-204* rescues HFD-induced impairment of endothelium-dependent vasorelaxation. *n* represents the number of aortic rings. NPD-SC: *n*=5, HFD-SC: *n*=10, HFD-204-I: *n*=10, *P<0.05 versus NPD-SC. ^#^*P*<0.05 versus HFD-SC. A, acetylcholine; *eSirt1*^*−/−*^, mice conditionally lacking endothelial *Sirt1*; HFD, high-fat diet; L, lumen; NPD, normal pellet diet; PE, phenylephrine. Independent sample *t*-test was used. Data shown as mean and error bar represents s.e.m.

We then asked whether vascular *miR-204* plays a causative role in endothelial dysfunction and vascular inflammation secondary to HFD feeding. Locked nucleic acid anti-*miR-204* was systemically delivered with osmotic mini-pumps for the final 6 weeks of the 8-week HFD feeding period, resulting in robust suppression of *miR-204* in mouse aortas (Fig. 4a; Supplementary Fig. 9a), but no change in weight gain, fat mass or blood glucose (Supplementary Fig. 9b–d). *In vivo* delivery of anti-*miR-204* prevented HFD-triggered downregulation of aortic Sirt1 (Fig. 4b) and HFD-induced decline of endothelium-dependent vasorelaxation (Fig. 4c) and bioavailable NO (Fig. 4d). In addition, HFD-induced vascular inflammation, as assessed by aortic macrophage content, was reduced in mice receiving anti-*miR-204* (Fig. 4e,f). Thus, *miR-204* mediates impaired endothelial function and vascular inflammation secondary to a HFD.

Systemic delivery of *miR-204* inhibitor may affect expression of non-vascular *miR-204* targets. We therefore examined the effect of *miR-204* inhibition on isolated aortas. *Ex vivo* inhibition of *miR-204* with anti-*miR-204* in aortas of mice subjected to HFD feeding improved endothelium-dependent vasorelaxation (Fig. 4j), indicating that the extravascular effects of *miR-204* inhibition do not play a major role in improving vascular endothelial function.

### *MiR-204* acts primarily via endothelial Sirt1

Because microbiome-dependent changes in vascular *miR-204* and Sirt1 expression were not limited to the endothelium (Figs 2a–g and 3a–f), we more carefully gauged the role of endothelial Sirt1 in improving endothelial function rendered by *miR-204* inhibition. Lack of endothelial Sirt1 (*eSirt1*^*−/−*^ mice) did not completely negate the effect of *miR-204* inhibition on HFD-induced endothelial dysfunction, despite a marked decrease in aortic *miR-204* (Fig. 4g,h). However, comparison of overall impairment of endothelium-dependent vasorelaxation in wild-type and *eSirt1*^*−/−*^ mice indicated that the absence of endothelial Sirt1 significantly diminishes the beneficial impact of *miR-204* inhibition on vascular endothelial function (Fig. 4i). Thus, endothelial Sirt1 is a key but not the only target of *miR-204* in HFD-induced endothelial dysfunction. Given the finding that neither antibiotics nor *miR-204* inhibition impacted endothelium-independent vasorelaxation (Supplementary Fig. 12), it is likely that targets of miR-204, other than *Sirt1*, that are partly responsible for *miR-204*-mediated endothelial dysfunction with HFD feeding are also expressed in the endothelium.

### Microbiome-dependent serum factor(s) regulate *miR-204*

Remote regulation of endothelium-dependent vasorelaxation by the microbiome suggests the presence of second messenger(s) in the systemic circulation that govern expression of vascular *miR-204* and Sirt1. Supporting this line of thought, endothelial cells incubated with serum from mice in which the gut microbiome was suppressed with antibiotics showed decreased *miR-204* and *Trpm3*, and increased Sirt1, expression (Supplementary Fig. 10a–c). In contrast, serum from mice in which the microbiome was repopulated by discontinuation of antibiotics did not downregulate *miR-204* in endothelial cells (Supplementary Fig. 10d). In addition, serum from antibiotic-treated mice led to the increase in endothelial Stat3 signalling (Supplementary Fig. 10a).

The effect of the microbiome on atherosclerosis, glucose homeostasis and adiposity has been attributed, in part, to circulating metabolites such as trimethylamine-oxide and short-chain fatty acids (SCFAs) produced by gut bacteria^2^,^3^,^4^,^5^,^7^,^20^. The receptors for SCFAs, including Gpr41 and 43, and Olfr78, are expressed in the vasculature such as the aorta^21^, renal arteries^21^ and myometrial arteries^22^. We tested whether SCFAs serve as second messengers responsible for the effect of the microbiome on aortic *miR-204* and Sirt1 expression, and endothelial function. Supplementation of drinking water with acetate and butyrate, two SCFAs produced by gut bacteria, did not negate the effect of antibiotics on aortic *miR-204* expression and endothelium-dependent vasorelaxation (Supplementary Fig. 11a,b). In addition, a mixture of SCFAs did not decrease Sirt1 in endothelial or smooth muscle cells *in vitro* (Supplementary Fig. 11c,d). The lack of effect of SCFAs suggests that they are not responsible for the communication between the gut microbiome and vascular *miR-204*.

**Figure 5:**
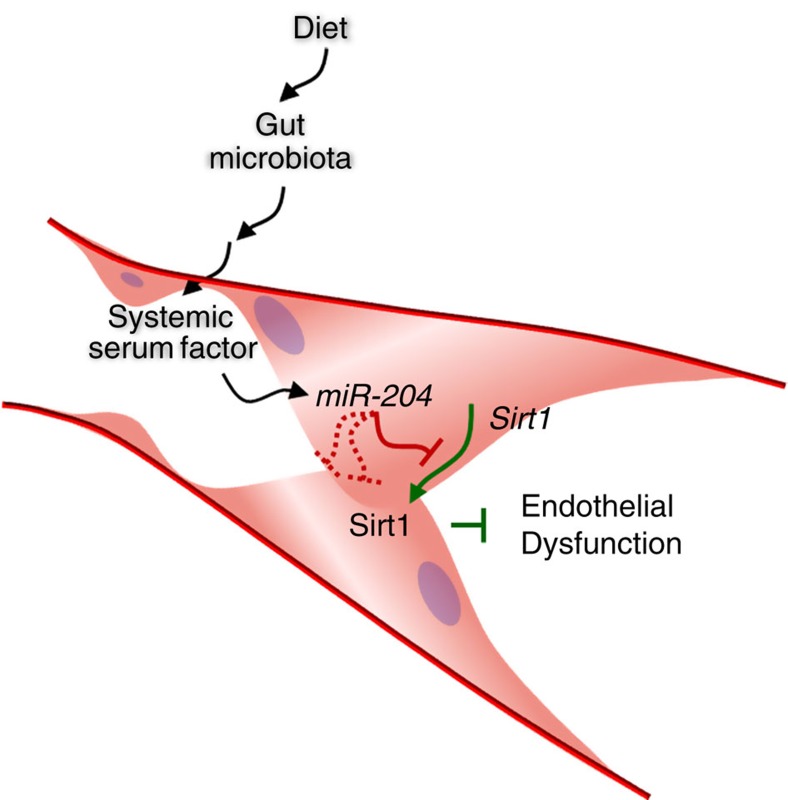
Remote regulation of vascular endothelial function by the microbiome. Gut microbiota-regulated second messengers in the systemic circulation induce vascular *miR-204* expression which, in turn, promotes endothelial dysfunction by targeting Sirt1.

In summary, these findings identify a gut–vascular axis in which microbes remotely upregulate *miR-204* expression in the vessel wall. Among the genes targeted by *miR-204* in the vascular wall is *Sirt1*, downregulation of which promotes endothelial dysfunction (Fig. 5). In addition, this work demonstrates that nutritional stress in the form of a western diet negatively impacts the endothelium via this same gut–vascular axis. Although the data do not identify the mediator(s) responsible for this remote communication between the gut and the vasculature, they do suggest that these messengers are in the systemic circulation, and regulate endothelial *miR-204* expression through the Stat3 transcriptional program. It is important to acknowledge that while orally administered antibiotics led to a striking diminution of the gut flora, because a fraction of these antibiotics may be systemically absorbed, their effect on vascular *miR-204*/Sirt1 expression and endothelial function could also be due to their impact on the composition of the microbiome in niches other than the gut. Finally, given that microbiome-dependent expression of *Sirt1* was not limited to the vasculature, and *miR-204* was not the only microRNA differentially regulated in the vasculature in germ-free conditions, these findings may provide a broader paradigm for microRNA-mediated regulation of organ physiology and pathophysiology by the microbiome.

## Methods

### Animals

All animal experiments were approved by Institutional Animal Care and Use Committee and were carried out according to National Institutes of Health (NIH) guidelines. Male GFM and PFM (Swiss-Webster, age 10 weeks) were procured from Taconic, New York, USA. All other studies were performed in C57BL/6, *Sirt1*^*fl/fl*^, *VE-Cadherin-Cre* and endothelium-specific *Sirt1* knockout (*eSirt1*^*−/−*^) mice (age 10–12 weeks). *eSirt1*^*−/−*^ mice were generated by crossing *Sirt1*^*fl/fl*^ mice with VE-Cadherin-Cre mice. Mice were given either normal pellet diet (NPD) or HFD. HFD is an adjusted calorie diet that provides 42% calories from fat (TD.88137, Harlan). To suppress the gut flora, mice were removed from the colony, housed separately and were given water supplemented with a cocktail of broad-spectrum antibiotics (metronidazole—1 g l^*−*1^, ampicillin—1 g l^*−*1^, neomycin—1 g l^*−*1^ and vancomycin—0.5 g l^*−*1^) for a period of 6 weeks. To repopulate antibiotic-treated mice with microbiota, antibiotic water was replaced with normal water and mice were co-housed with their former littermates for a period of 2–3 weeks. Mice were maintained in specific pathogen-free conditions at the central animal facility of the University of Iowa. The mice receiving cocktail of broad-spectrum antibiotics were monitored for weight loss, diarrhoea and signs of discomfort. Suppression of gut microbiota was confirmed by 16S RNA gene analysis. SCFAs (acetate 90 mM; butyrate 60 mM) were added to drinking water. These concentrations were based on the published work^23^. Control mice received sodium-matched water. For *in vivo* infusion of locked nucleic acid microRNA inhibitor, ALZET 2006 osmotic pups containing oligonucleotides (scrambled control or *miR-204* inhibitor (*miR-204-I*)) were aseptically implanted in C57BL/6 or *eSirt1*^*−/−*^mice kept on either NPD or HFD. Mini-osmotic pumps were designed to deliver oligonucleotides at the rate of 0.15 μl h^*−*1^, and each mouse received oligonucleotides at a dose of ∼0.7 mg kg^*−*1^ per day for a period of 6 weeks.

### Reagents

MicroRNA cDNA preparation kit and primers were purchased from Quanta Biosciences Inc., Gaithersburg, MD, USA. Sirt1 (Thermo Scientific-PA5-23-063, Santa Cruz Biotechnology-sc-15404), Gapdh (Trevigen-2275-PC), eNOS (BD Transduction Laboratories-610297), Stat3 (Cell Signalling Technology-4904), phospho- (Tyr-705)-Stat3 (Cell Signalling Technology-9145), F4/80 (abcam-ab6640) antibodies were used.

### RNA isolation and RT–qPCR

RNA was isolated using Qiazole/Trizole as per the manufacturer's instructions. MicroRNAs were converted to cDNA using the qScriptTM microRNA cDNA synthesis kit (Quanta Biosciences). Real-time qPCR was performed using Brilliant II SYBR Green RT–qPCR kit. *MiR-29b*, actin (beta), *Rnu6* or *Snord47* was used as internal controls. qPCR with reverse transcription (RT–qPCR) of *Sirt1*, *Trpm3* and *Gapdh* were performed using the Brilliant II SYBR Green RT–qPCR kit. *Gapdh* was used as an internal control. Primer sequences are provided in Supplementary Table 1.

### NanoString nCounter assay and selection of internal control for qPCR

Total aortic RNA was used as input for the nCounter reactions and was performed according to the manufacturer's instructions. In brief, a unique oligonucleotide was tagged to the microRNAs for the specific and sensitive detection by nCounter analysis system. The system is based on target-specific probe pairs that are hybridized to sample in solution. A reporter probe carries the fluorescent signal, while capture probe allows the complex to be immobilized for data collection. The protocol does not involve any amplification steps. The panel was designed to screen 578 mouse microRNAs, 33 viral microRNAs, 3 non-mammalian miRNAs, 8 negative controls and a dilution series of 6 positive controls. nCounter data were normalized for lane-to-lane variation by six dilution series spike positive controls. Global microRNA expression was used to normalize individual microRNA expression. Heat map for expression of microRNAs was prepared using GenePattern (http://genepattern.broadinstitute.org/gp/pages/index.jsf). In the absence of universally accepted internal control for normalization of aortic microRNA expression analysis by qPCR, we identified a microRNA that best resembles global microRNA expression as previously described^24^ with some modifications. In brief, to select the microRNA most suitable as a normalization control, the pattern of microRNA expression in PFM and GFM mice normalized with (i) candidate microRNAs and (ii) commonly used normalization mRNAs (*Actb (β-actin*), B2m *(β-2-microglobulin*) and *Gapdh*) was determined and compared with expression pattern as achieved with normalization with global microRNA expression. The data were clustered by pairwise complete linkage based on Pearson correlation using GenePattern (http://genepattern.broadinstitute.org/gp/pages/index.jsf). The normalization of microRNAs by *miR-29b* was found to best resemble the expression profile as achieved with normalization using global microRNA expression, followed by *miR-29c*, *β-actin* and *Gapdh* (Supplementary Fig. 2a), and thus *miR-29b* was used as an internal control for qPCR experiments in aortic samples. To ascertain change in the expression and to validate selection of internal control for qPCR, a single microRNA (*miR-204*) was normalized by another common housekeeping gene beta-actin, and the expression of multiple microRNAs was normalized with *miR-29b*, to see if the pattern observed with nCounter assay remains the same (Supplementary Fig. 2b–e).

### Target gene prediction

MicroRNA-SVR is an algorithm that ranks microRNA–mRNA target sites interaction (SVR score). Lower SVR score indicates stronger interaction between a microRNA and mRNA target site^25^. Differentially regulated microRNAs in GFM were examined for their target genes based on the SVR score (www.microRNA.org, the data are available as tab delimited files). SVR scores of each microRNA for every targeted gene were tabulated at different binding strengths (threshold SVR score <−0.5, <−0.7 and <−0.9), and number of microRNAs targeting a specific gene was determined. Several microRNAs targeting a specific gene were predicted to be differentially regulated.

### *In vitro* and *ex vivo* transfection of oligonucleotides

Oligonucleotides were incubated with Lipofectamine2000 (Invitrogen) at room temperature for 20 min before adding to cells in OPTI-MEM (Invitrogen). Four hours later, the medium was replaced by fresh medium and cells were cultured for an additional specified period of time (24–72 h). *Ex vivo* transfection of oligonucleotides into mouse thoracic aorta was performed using Oligofectamine (InVitrogen). Oligos were incubated with Oligofectamine at room temperature for 20 min before adding to aortas in OPTI-MEM (InVitrogen). The aorta was kept in transfection mixture overnight. The sequence of oligonucleotides used in study is provided in Supplementary Table 1.

### Luciferase reporter assay

The pRL-CMV and Luciferase-Sirt1-3′-UTR plasmids were transfected in the human umbilical vein endothelial cells (HUVECs) with scrambled microRNA or *miR-204* mimic (20 pM). Lipofectamine 2000 was used as a transfection agent. The luciferase-to-renilla ratio was calculated to normalize for variations in transfection efficiencies.

### *Ex vivo* adenoviral infections

Gene transfer was achieved *ex vivo* by incubating aortas from mice with 5 × 10^8^ p.f.u. of the appropriate adenoviral stock at 37 °C for 24 h before recording vascular reactivity. For *in vitro* infections of HUVECs, 100 multiplicity of infection was used. The viruses used were (i) Ad*LacZ* coding for *Escherichia coli* LacZ, (ii) Ad*Sirt1* coding for Sirt1 and (iii) Ad-DN*Stat3* coding for non-phosphorylatable dominant-negative Stat3.

### Vascular reactivity

Thoracic aortas of mice were used. The animals were rapidly killed by CO_2_ inhalation. The aorta was carefully dissected, rapidly removed and placed in ice-cold oxygenated Krebs-Ringer bicarbonate solution. The vessel was carefully cleared of loose connective tissue and cut into 5–10 1.5 mm rings. A single ring from each mouse was suspended between two wire stirrups (150 μm) in a12.5- ml organ chambers of myograph system (DMT Instruments, FL, USA) in 6 ml Krebs-Ringer (95% O_2_–5% CO_2_, pH 7.4, 37 °C). One stirrup was connected to a three-dimensional micromanipulator, and the other to a force transducer. The mechanical force signal was amplified, digitalized and recorded (PowerLab 8/30). All concentration–effect curves were performed on arterial rings beginning at their optimum resting tone. This was determined by stretching arterial rings at 10 min intervals in increments of 100 mg to reach optimal tone (∼500 mg). One dose of KCl (60 mM) was administered to verify vascular smooth muscle viability. Vascular NO bioavailability was determined as the difference in cumulative dose–response curve for phenylephrine (PE; 10^*−*9^–10^*−*5^ M) in the absence or presence of the NO synthase inhibitor L-NAME. Data are presented as per cent change to the maximum contraction induced by PE (10^*−*5^ M). Endothelium-dependent and -independent vasorelaxation was determined by generating dose–response curves to acetylcholine (10^*−*9^–10^*−*5^ M) and sodium nitroprusside (10^*−*9^–10^*−*5^ M), respectively, on PE (10^*−*6^ M)-induced pre-contracted vessels. Vasorelaxation evoked by acetylcholine and sodium nitroprusside was expressed as per cent relaxation, determined by calculating the percentage of inhibition to the pre-constricted tension. Aortic rings not responding to initial dose of KCL or showing auto-relaxation were excluded. Per cent impairment in acetylcholine-induced vascular relaxation was determined by measuring the difference between the mean area under the curve for the NPD-scrambled control group and that of the test groups.

### NO_*x*_ (nitrate/nitrite) measurement

Aortic rings were carefully isolated and immediately frozen. Aorta (5–7 mm) was used for the nitrate/nitrite assay and was performed as per the manufacturer's instructions (Cayman-780001, Ann Arbor, MI, USA).

### Immunoblotting

Protein samples were resolved on 10–12% SDS–polyacrylamide gel electrophoresis and transferred to nitrocellulose membranes. Antigen–primary antibody complexes were incubated with horseradish peroxidase-conjugated secondary antibodies and visualized by western blotting luminol reagent (Thermo, USA). Images were captured and quantified using the Image Lab (BioRad, USA) software, and intensity values were normalized to Gapdh. Uncropped blots are shown in Supplementary Fig. 13.

### Histological processing and immunostaining

Sections (5 μm) of formalin-fixed paraffin-embedded tissues were heated (95 °C, 20 min) in citrate buffer (10 mM) for antigen retrieval, followed by incubation with primary antibodies. The working concentration for the primary antibodies were as follows; Sirt1; 1: 200, p-Stat3; 1: 100, vWF; 1:200, anti-f4/80; 1:200. Antigen–primary antibody complexes were probed with polyvalent biotinylated goat anti-rabbit secondary antibody and streptavidin–horseradish peroxidase system to amplify the signals, followed by detection with diaminobenzidine as a chromogen. Slides were counterstained with haematoxylin, dehydrated with alcohols and xylene, and mounted in DPX. Alternatively, antigen–primary antibody complexes were probed with fluorescence-tagged secondary antibodies. Aortic root cryosections (20 μM) were examined for anti-F4/80 staining using goat anti-rat secondary antibody and detection with diaminobenzidine. Images were captured using Zeiss confocal microscope (Model 710). Total aortic Sirt1 expression was determined by measuring fluorescence intensity using the ImageJ software. To examine the change in endothelial expression of Sirt1, endothelial cells were cropped from the images based on the vWF staining using the Photoshop CC software, and the intensity was determined using the ImageJ software.

### *In situ* hybridization

Deparaffinized and rehydrated aortic sections (6 μm) were treated with proteinase K (10 μg ml^−1^, 5 min, 37 °C). Sections were dehydrated using ethyl alcohol and incubated with pre-hybridization buffer, followed by hybridization mixture containing either double digoxigenin(DIG)-tagged *miR-204-5p* probe or DIG-tagged scrambled probe (36 h, 57 °C). Sections were washed with sodium saline citrate and probed with either Dylight 594-labelled anti-DIG goat antibody (working concentration; 1:500, Vector Laboratories, Burlingame, CA, USA) or alkaline phosphatase-conjugated anti-DIG FAB (fragment, antigen-binding) (18 h, 4 °C). Following alkaline phosphatase-conjugated FAB binding, BCIP/NBT (5-bromo-4-chloro-3′-indolylphosphate/nitro-blue tetrazolium) was used as a chromogen. Slides were counterstained with either Nuclear Fast Red or 4,6-diamidino-2-phenylindole and dehydrated with alcohol and mounted in DPX. Images were captured by charge-coupled device camera attached to the Zeiss microscope (Model BX61). For quantification, slides were counterstained with 4,6-diamidino-2-phenylindole. Six images originating from at least two aortic rings were used for the quantification. The blue–purple colour range was selected using the Photoshop CS6, converted to grey and quantified using the Image J software. Sequence of *miR-204-5p* double DIG-tagged probe is provided in Supplementary Table 1.

### Cell culture

HUVECs (Cat# CC-2519) were purchased from Clonetics (San Diego, CA USA) and cultured in growth factor- and fetal bovine serum-supplemented EGM-2 (Lonza, Walkersville, MD, USA). To examine the effects of mouse serum, 2% fetal bovine serum in EGM-2 was replaced with pooled serum from the specific group of mice.

### Statistical analysis

To determine sample sizes, power analysis was carried out using preliminary data sets. Considering the independent sample *t*-test, a sample size of 5 achieved >90% power for studies involving gene and protein expression. However, for vascular reactivity experiments, a sample size of 10 achieved >95% power to reject null hypothesis at the significance level of 0.05. All the data meet assumptions of the statistical test and different groups had similar variance. We performed all statistical analysis with SPSS (Version 17.0) unless specified. Significance of difference between two groups was evaluated using independent sample *t*-test. Results were considered significant if *P* values were ≤0.05.

### Data availability

Microarray data were deposited in the Gene Expression Omnibus database (Accession code GSE64630). The authors declare that the data supporting the findings of this study are available within the article and from the authors on request.

## Additional information

**How to cite this article:** Vikram, A. *et al.* Vascular *microRNA-204* is remotely governed by the microbiome and impairs endothelium-dependent vasorelaxation by downregulating Sirtuin1. *Nat. Commun.* 7:12565 doi: 10.1038/ncomms12565 (2016).

## Supplementary Material

Supplementary Information Supplementary figures 1-13, Supplementary table 1
