# Supplementary material for: Vascular microRNA-204 is remotely governed by the microbiome and impairs endothelium-dependent vasorelaxation by downregulating Sirtuin1
Source: Nat Commun. 2016 Sep 2;7:12565. doi: 10.1038/ncomms12565 (PMC5025761; doi:10.1038/ncomms12565)
Supplement: Supplementary Information — Supplementary figures 1-13, Supplementary table 1 [file ncomms12565-s1.pdf]

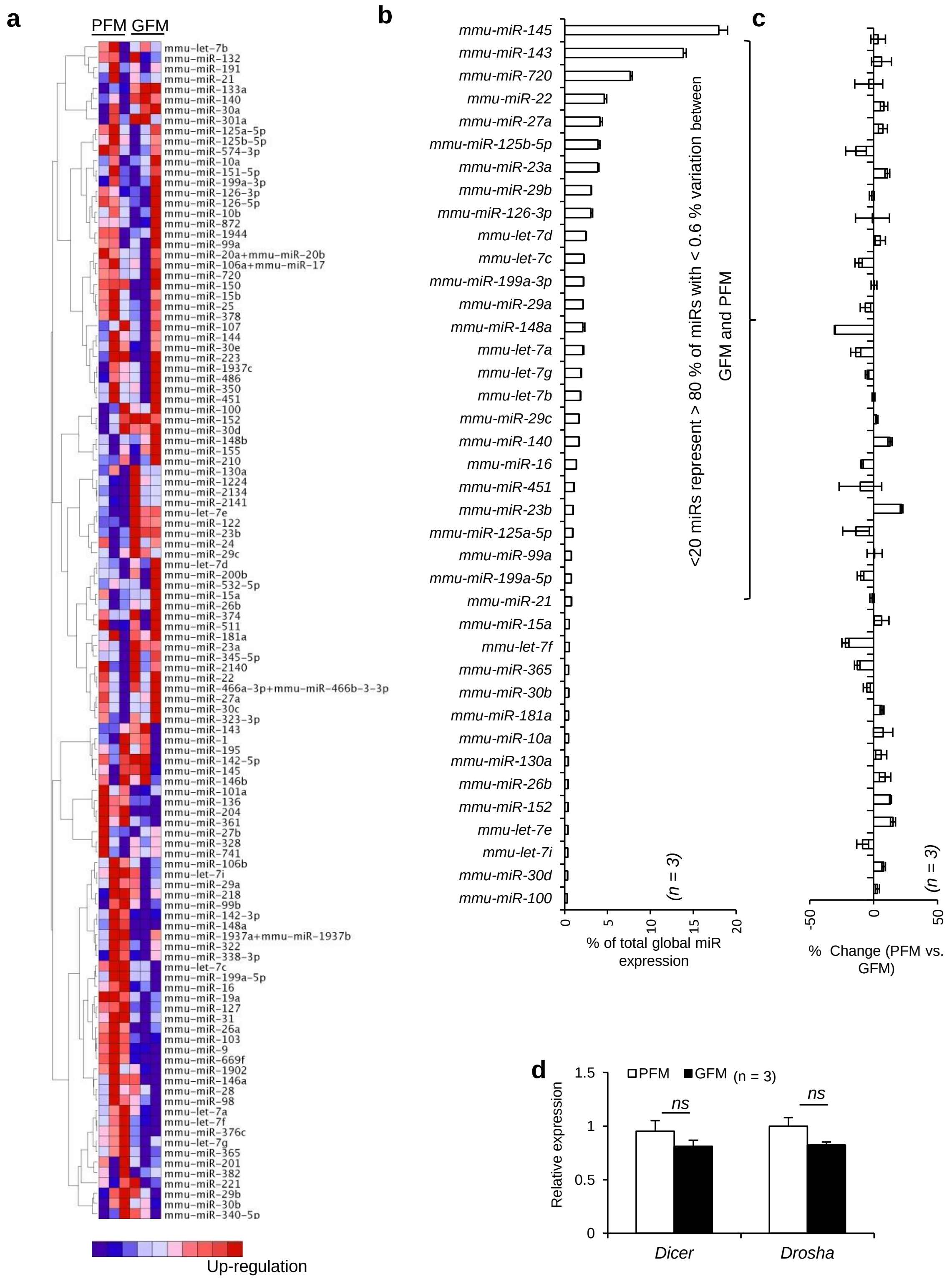

Supplementary Fig. 1. Aortic microRNAs are differentially expressed in PFM v. GFM. (**a**) Heat map of differentially regulated microRNAs in aortas of PFM and GFM. Each column represents one mouse. n = 3 in each group. (**b**) Percent expression of most abundant microRNAs in aortas of PFM. n = 3 for each microRNA. (**c**) Percent change in expression of most abundant microRNAs in aortas of GFM compared to PFM. n = 3 for each microRNA. (**d**) Relative expression of Dicer and Drosha in aortas of GFM and PFM. n = 3 for each group. *ns*: not significant by independent sample t-test. Data shown as mean and error bar represents s.e.m.

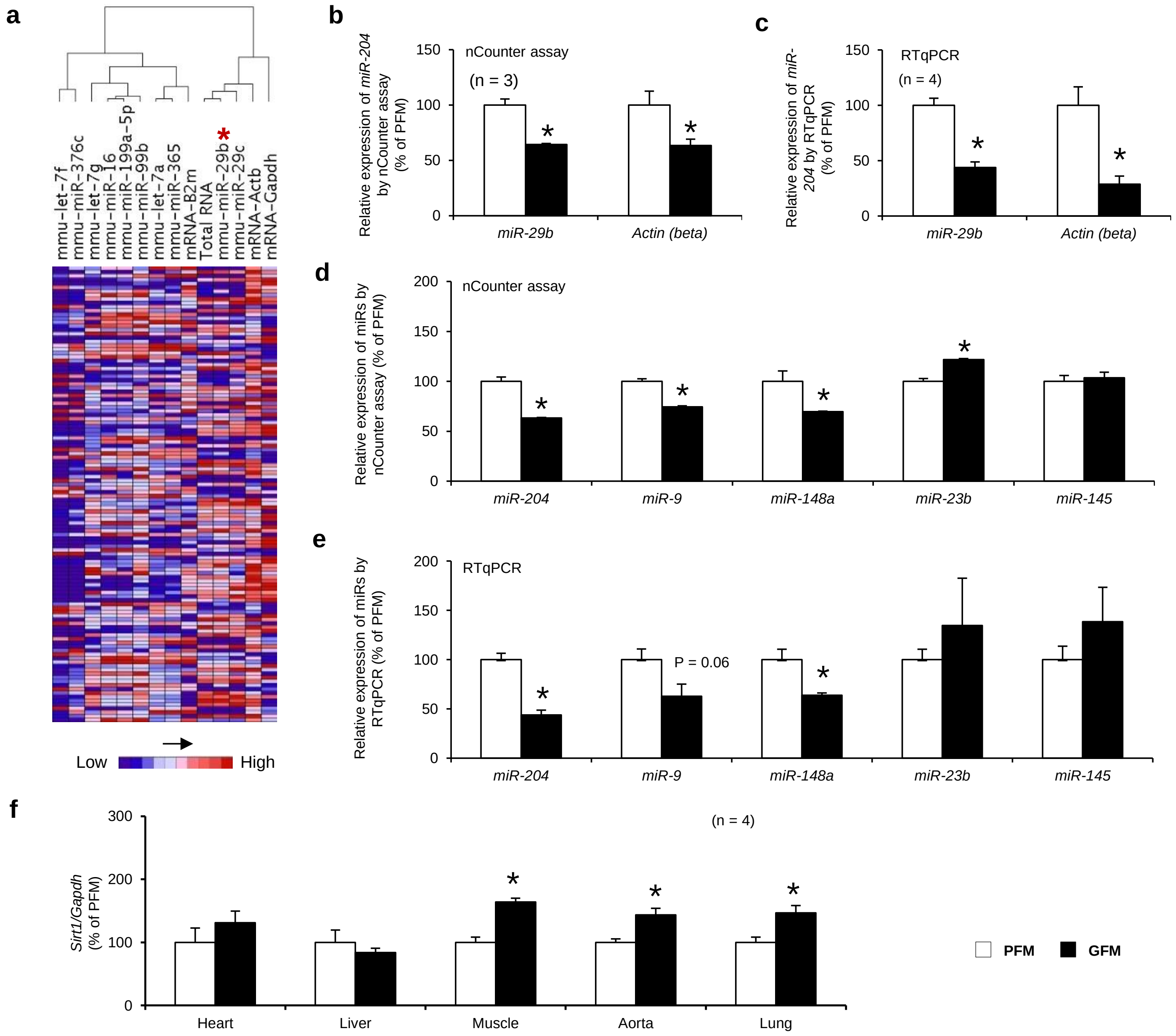

Supplementary Fig. 2. *MiR-204* is downregulated in aortas of GFM. (a) Heat map of nCounter array showing microRNA expression pattern normalized to candidate microRNAs, commonly used control mRNAs, and global microRNA expression (mmu-T-microRNAs). Each row represents a microRNA. Data were clustered by pairwise complete linkage based on Pearson correlation. Data shows that using *miR-29b* (red asterisk) as the internal normalization control produced a microRNA expression profile that most closely resembled the expression profile when using global microRNA (Total miRNA) as the normalization control. (**b-c**) *miR-204* expression measured by nCounter array (**b**) and qPCR (**c**) in aortas of GFM and PFM, normalized to *microRNA-29b* and *β-Actin* as internal controls. *p < 0.05 vs. PFM. (**d**) Expression of *miR-204, miR-9, miR-148a, miR-23b and miR-145* measured by nCounter assay in aortas of GFM and PFM normalized to global miRNA. (**e**) Expression of *miR-204, miR-9, miR-148a, miR-23b* and *miR-145* by qPCR in aortas of GFM and PFM normalized to *miR-29b*. *p < 0.05 vs. PFM. The selected microRNAs represent down-regulated (*miR-204, miR-9 and miR-148a*), an upregulated (*miR-23b*), and a highly expressed microRNA with no change in expression (*miR-145*). (**f**) *Sirt1* expression in heart, liver, aorta, skeletal muscle, and lungs of GFM and PFM. n = 4 for each group, *p < 0.05 vs. PFM. Independent sample t-test was used. Data shown as mean and error bar represents s.e.m.

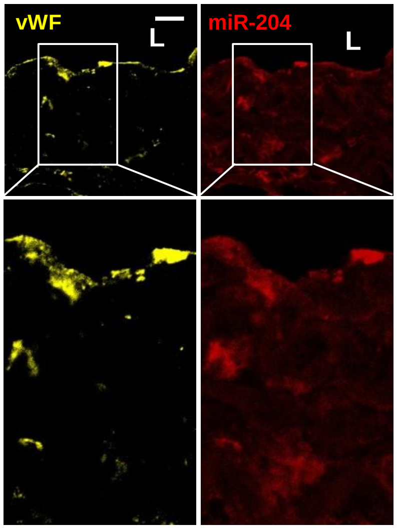

Supplementary Fig. 3. *miR-204* is expressed in vascular endothelium and tunica media. Immunofluorescence for *miR-204* in mouse aortas. vWF: von Willebrand factor. L: lumen. (magnification ×63, Scale bar; 20μm).

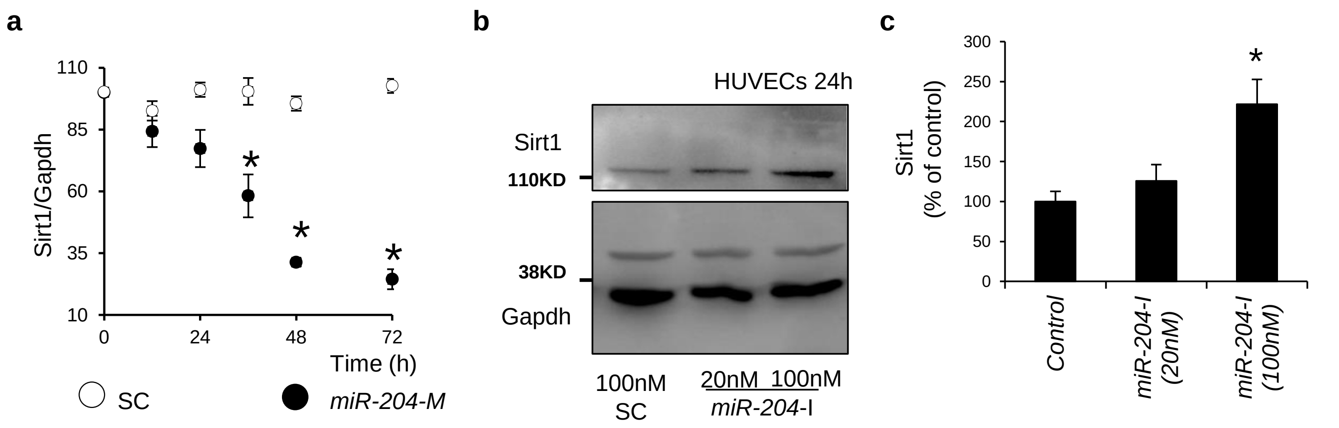

Supplementary Fig. 4. *miR-204* regulates Sirt1 expression in endothelial cells. (**a**) *miR-204* mimic (*miR-204*-M) suppresses Sirt1 expression in HUVECs in time dependent manner. n = 3 independent experiments. *p < 0.05 vs. SC. (**b**) *miR-204* inhibitor (*miR-204*-I) upregulates Sirt1 expression in HUVECs. SC: scrambled control microRNA. Representative of 3 independent experiments. (**c**) Quantification of Sirt1 expression in b. n = 3. *p < 0.05 vs. SC. Independent sample t-test was used. Data shown as mean and error bar represents s.e.m.

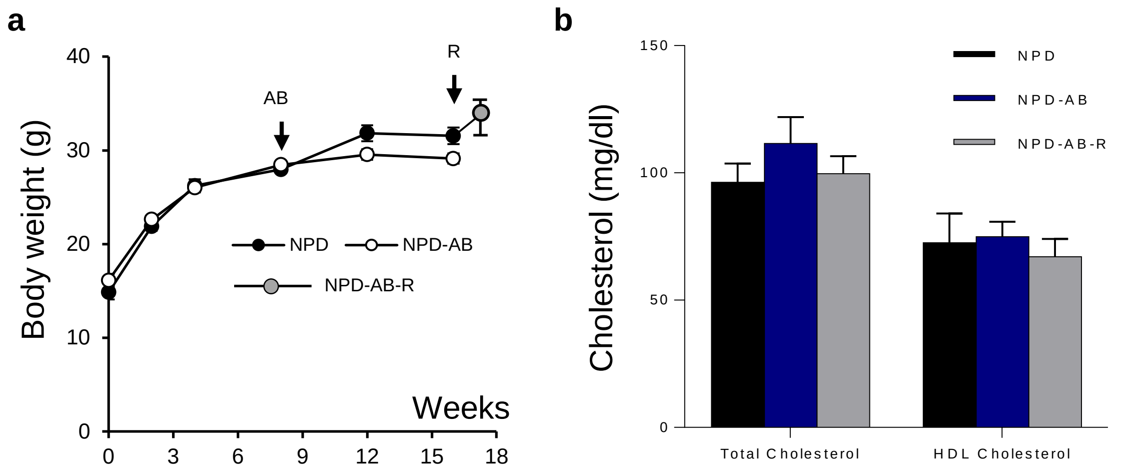

Supplementary Fig. 5. Oral broad-spectrum antibiotics slightly decrease body weight and do not affect plasma lipids. (**a**) Effect of broad-spectrum antibiotics on mouse body weight. Arrowheads indicate isolation of mice from the colony and initiation of antibiotics in drinking water (AB), and discontinuation of antibiotics together with co-habitation of the mice with littermates not on antibiotics (AB-R). n = 6 for NPD and NPD-AB; n = 3 for NPD-AB-R. (**b**) Effect of antibiotics on total cholesterol and HDL-Cholesterol. n = 6 for NPD and NPD-AB; n = 3 for NPD-AB-R. NPD: normal pellet diet; AB: Antibiotics; R: stoppage of antibiotics + co-habitation. Data shown as mean and error bar represents s.e.m.

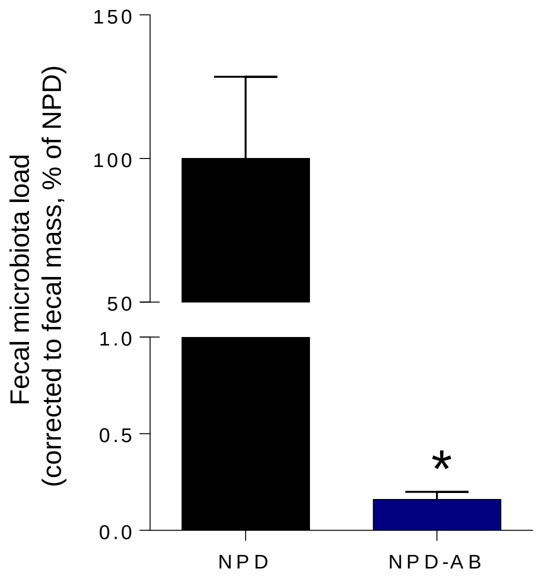

Supplementary Fig. 6. Oral broad-spectrum antibiotics suppress fecal microbial load. Fecal microbial load was measured by qPCR of bacterial 16S RNA in mice on normal pellet diet (NPD) or mice on NPD receiving broad-spectrum antibiotics for 6 weeks (NPD-AB). n = 5 in each group. *P < 0.05 vs. NPD by independent sample t-test. Data is shown as mean normalized to NPD and error bar represents s.e.m.

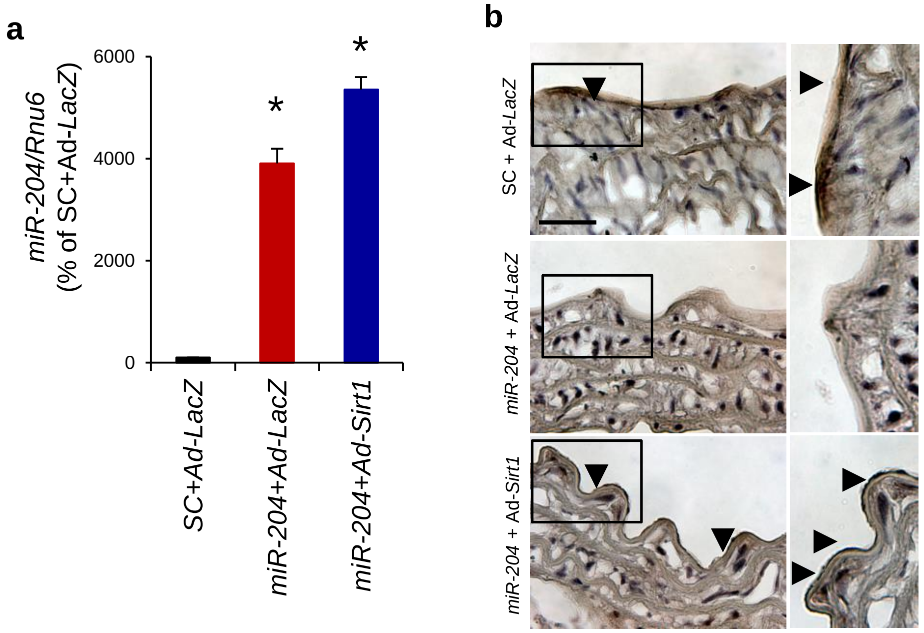

Supplementary Fig. 7. Ad-*Sirt1* rescues endothelial Sirt1 expression suppressed by *miR-204* mimic *ex vivo*. (a) *miR-204* expression in explanted mouse aortas transfected with *miR-204* mimic, followed by infection with Ad*Sirt1* or Ad*LacZ*. n = 4 aortic rings from 3 mice. *$p < 0.05$ *vs.* SC + AdLacZ by independent sample t-test. **(b)** Effect of *miR-204* mimic and/ Ad*Sirt1* or Ad*LacZ* on expression of endothelial Sirt1 in mouse aortas**.** Arrowheads indicate endothelial immunostaining for Sirt1 (Magnification ×100, Scale bar; 20 μm). SC: scrambled control microRNA; Ad*Sirt1*: adenovirus encoding *Sirt1*; Ad*LacZ*: control adenovirus encoding *E. Coli LacZ* gene. Data shown as mean and error bar represents s.e.m.

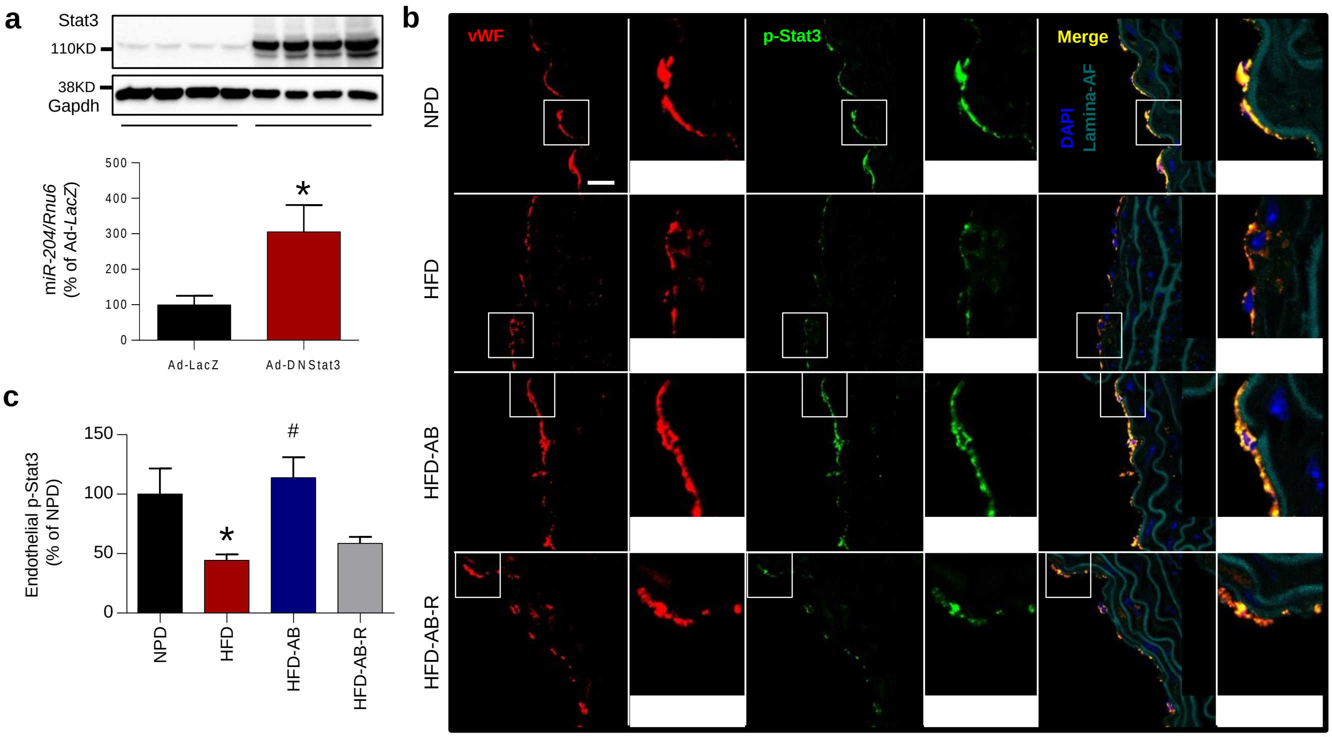

Supplementary Fig. 8. Endothelial Stat3 signaling inhibits *miR-204* expression, is suppressed by HFD, and rescued by broad-spectrum antibiotics. (**a**) Adenoviral mediated overexpression of dominant negative *Stat3* (Ad-DN*Stat3*) upregulates *miR-204* expression in HUVECs. n = 4 in each groups. *p < 0.05 vs. Ad*LacZ*. (**b**) Immunofluorescence staining (magnification ×63, Scale bar; 20 μm) of mouse aortic sections showing downregulation of endothelial phospho-Stat3 by HFD feeding, and rescue of endothelial STAT3 signaling with suppression of gut microbiota with broad-spectrum antibiotics. Effect of antibiotics on endothelial phospho-Stat3 was reversed with stoppage of antibiotics. Lamina-AF: laminar autofluorescence. (**c**) Quantification of endothelial p-Stat3 immunostaining in *b*. NPD: n = 5, HFD: n = 5, HFD-AB: n = 5 and HFD-AB-R: n = 3. *p < 0.05 vs. NPD, #P < 0.05 *vs.* HFD. HFD: High-fat diet; NPD: normal pellet diet; AB: Antibiotics; R: stoppage of antibiotics + co-habitation. Independent sample t-test was used. Data shown as mean and error bar represents s.e.m.

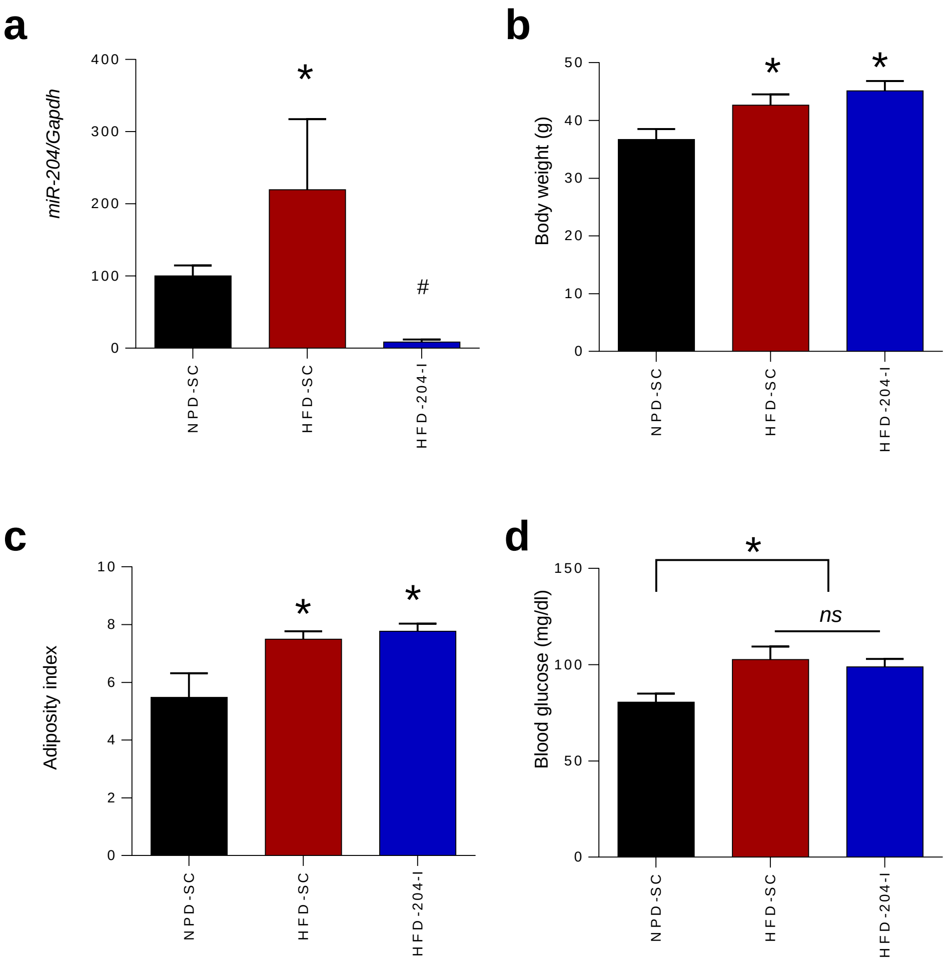

Supplementary Fig. 9. Systemic inhibition of *miR-204* does not affect body weight, adiposity, and blood glucose. (a) *miR-204-I* suppresses *miR-204* expression in mouse aortas. (**b-d)** *miR-204-I* does not affect body weight (**b**), adiposity (**c**), and fasting blood glucose (**d**). *$p < 0.05$ vs. NPD, #$P < 0.05$ *vs.* HFD. n = 4 for NPD-SC, n = 6 for HFD-SC and HFD-204-I. NPD: normal pellet diet; HFD: high-fat diet; SC: scrambled control microRNA. Independent sample t-test was used. Data shown as mean and error bar represents s.e.m.

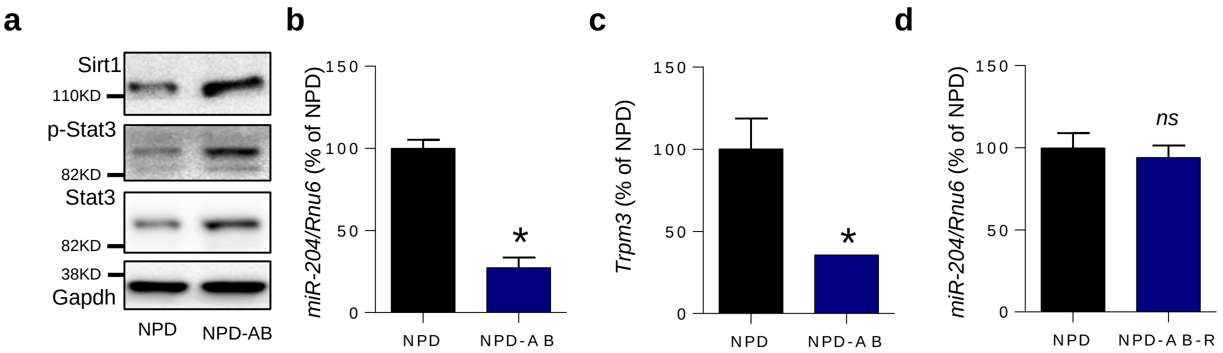

Supplementary Fig. 10. Serum of mice on broad-spectrum antibiotics promotes endothelial Stat3 signaling and Sirt1, and inhibits endothelial *miR-204* expression. (a-c) Serum of mice on broad-spectrum antibiotics upregulates Sirt1 and phospho-Stat3 **(a)**, and downregulates *miR-204* and *Trpm3* (**b, c**) in HUVECs. (**d**) Serum from mice in which antibiotics were discontinued does not suppress *miR-204* in HUVECs. n = 3 independent experiments, serum was pooled from NPD (n = 6) and NPD-AB (n = 4) and NPD-AB-R (n = 3) mice. *P < 0.05 *vs.* NPD. NPD: normal pellet diet; AB: Antibiotics; R: stoppage of antibiotics + co-habitation. Independent sample t-test was used. Data shown as mean and error bar represents s.e.m.

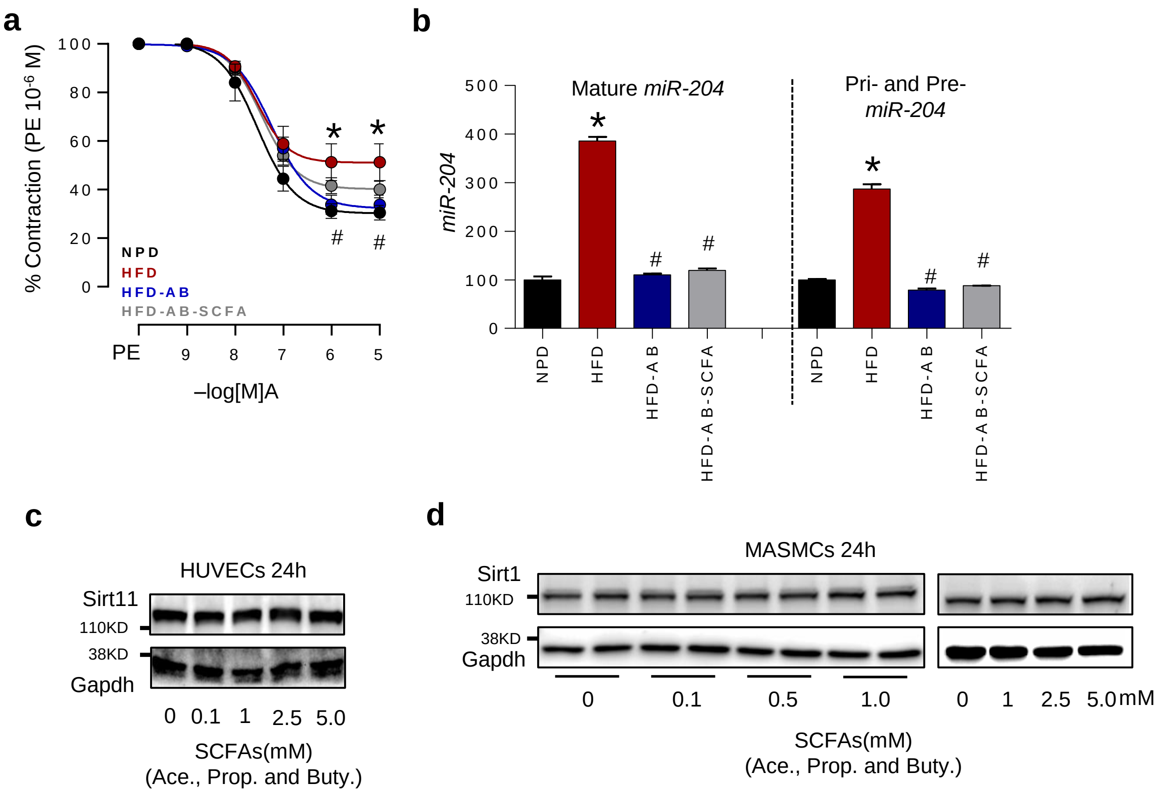

Supplementary Fig. 11. Short-chain fatty acids (SCFAs) do not negate the effect of antibiotics on endothelium-dependent vasorelaxation and aortic *miR-204* expression, and do not affect Sirt1 expression in endothelial or smooth muscle cells. (**a-c**) Supplementation of drinking water with SCFAs [sodium acetate (90mM) and sodium butyrate (60mM)] does not negate **(a)** improvement in endothelium-dependent vasorelaxation and **(b)** downregulation of mature and precursor *miR-204* in mouse aortas induced by suppression of gut microbiome by antibiotics in HFD-fed mice. n represents number of aortic rings in *a*. NPD: n = 7, HFD: n = 9, HFD-AB: n = 12, HFD-AB-SCFA: n = 18. *p < 0.05 vs. NPD. #p < 0.05 vs. HFD. PE: phenylephrine; A: acetylcholine. (**c-d**) Mixture of SCFAs does not affect Sirt11 expression in HUVECs (**c**) and mouse aortic smooth muscle cells (MASMCs) (**d**). Ace: acetate; Prop: propionate; Buty: butyrate. Independent sample t-test was used. Data shown as mean and error bar represents s.e.m.

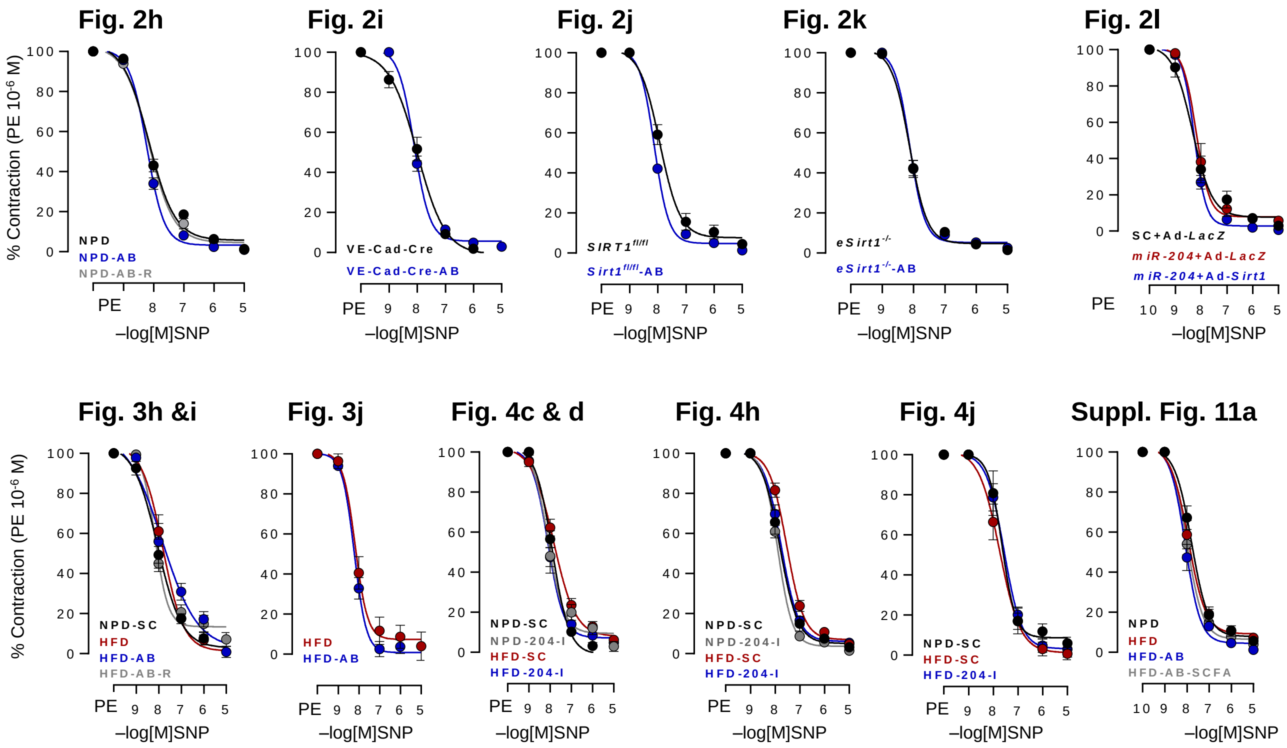

Supplementary Fig. 12. Sodium nitroprusside (SNP)-induced endothelium-independent vasorelaxation of the aorta is not regulated by the gut microbiome or *miR-204*. Data corresponds to experiments done for the indicated figures in the manuscript.

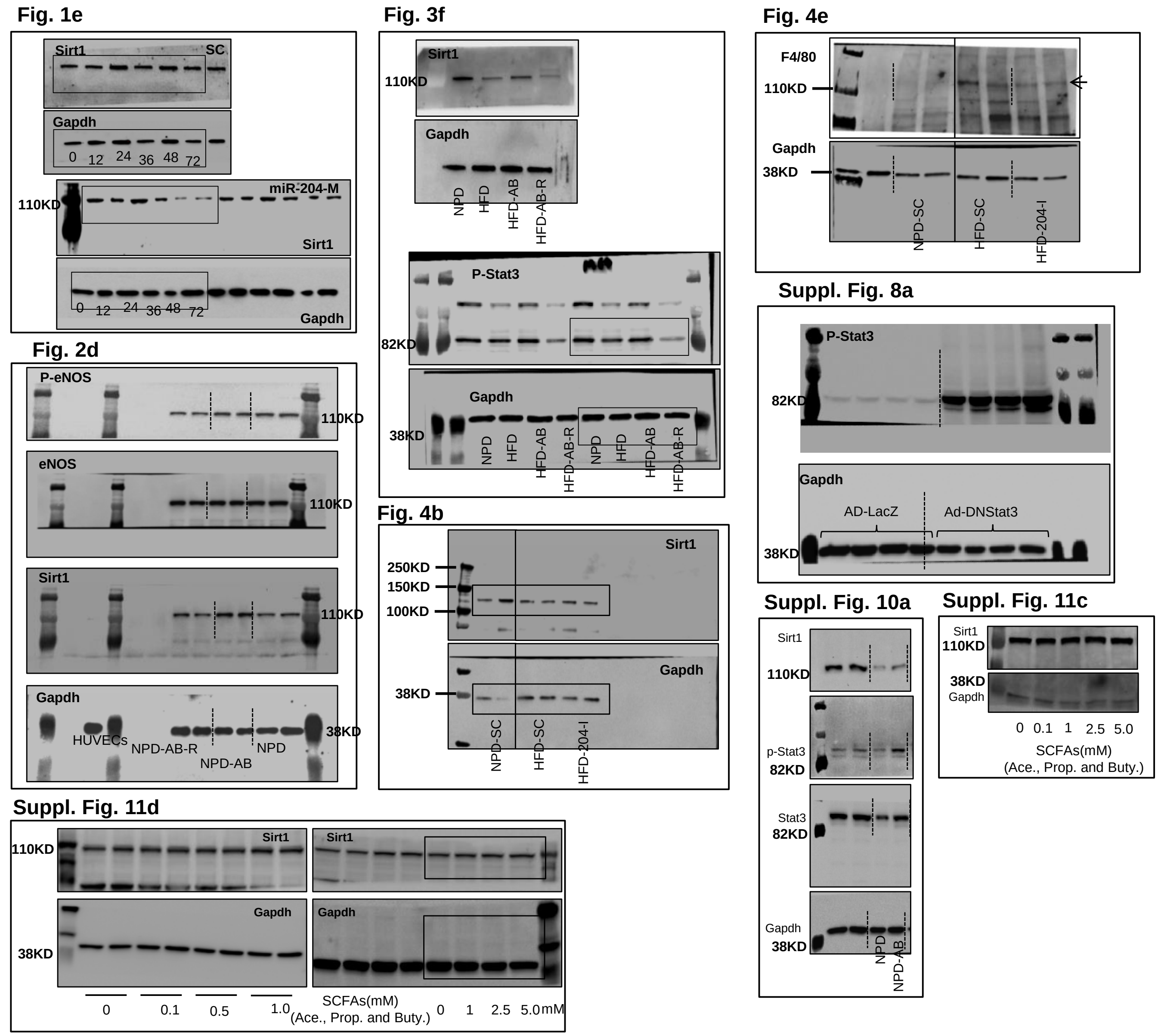

Supplementary Fig. 13. Original immunoblots for indicated figures.

Supplementary Table 1. Sequence of primers, mature microRNAs, detection probes and microRNA-mimic/inhibitor used in the study.

mRNA	Primer sequence	
	Forward	Reverse
Sirt1 (mouse)	5’-AAT GCT GGC CTA ATA GAC TTG CA-3’	5’-CCG TGG AAT ATG TAA CGA TTT GG-3’
Trpm3 (mouse)	5’-ACC CCG TCA AGT AGT G-3’	5’-CCC CAA AGT TGG CGT-3’
TRPM3(human)	5’-CGC AGC TGG AAG ACC TTA TC-3’	5’-AAG CTG CTC TGA CGG ACA AT-3’
Gapdh(mouse)	5’-GGC AAA TTC AAC GGC ACA GT-3’	5’-CGC TCC TGG AAG ATG GTG AT-3’
GAPDH (human)	5’-ATG ACA TCA AGA AGG TGG TG -3’	5’-CAT ACC AGG AAA TGA GCT TG-3’
eNOS (mouse)	5’-GAA GGG AAG TGC AGC AAA GG-3’	5’-CAG AGA TCT TCA CTG CAT TGG CTA-3’
Drosha (mouse)	5’-GGA CCA TCA CGA AGG ACA CT-3’	5’-CAC GGG TCT CTT GGT TTT GT-3’
Dicer (mouse)	5’-ACC AAG TGA TCC GTT TAC GC-3’	5’-CAA CCG TAC ACT GTC CAT CG-3’

microRNA	Mature microRNA sequence
miR-204-5p	5'-UUC CCU UUG UCA UCC UAU GCC U-3'
miR-29b	5'-UAG CAC CAU UUG AAA UCA GUG UU-3'
miR-145-5p	5'-GUC CAG UUU UCC CAG GAA UCC CU-3'
miR-148a-3p	5'-UCA GUG CAC UAC AGA ACU UUG U-3'
miR-23b-3p	5'-AUC ACA UUG CCA GGG AUU ACC-3'
miR-9-5p	5'-UCU UUG GUU AUC UAG CUG UAU GA-3'

microRNA primers were procured from Quanta Bioscience. miScript Precursor assay primer which detects stem-loop in both pri- and pre- *miR-204* was procured from QIAGEN.

Modulators	Sequence
SC	5’-ACG TCT ATA CGC CCA- 3’
miR-204-mimic	5'-UUC CCU UUG UCA UCC UAU GCC U-3'
miR-204-inhibitor	5’-AGG ATG ACA AAG GGA-3’
miR-204 in-situ hybridization probe	5’-Dig-N-AGG CAT AGG ATG ACA AAG GGA A-N-Dig-3’
Scrambled in-situ hybridization probe	5’-Dig-N-GTG TAA CAC GTC TAT ACG CCC A-N-Dig-3’
